# Supplementary material for: Tools for engineering coordinated system behaviour in synthetic microbial consortia
Source: Nat Commun. 2018 Jul 11;9:2677. doi: 10.1038/s41467-018-05046-2 (PMC6041260; doi:10.1038/s41467-018-05046-2)
Supplement: Supplementary file 1 — Supplementary Information [file 41467_2018_5046_MOESM1_ESM.pdf]

Tools for engineering coordinated system behaviour in synthetic microbial consortia

Supplementary Information

Supplementary Methods

Cell fluorescence measurements and flow cytometry data analysis

Cell fluorescence of *E. coli* TOP10 cells transformed with composite modules of AHL-receiver devices and the GFP reporter module was recorded by flow cytometry.

Following cell culture and induction as described in the Methods section of the main manuscript, cell culture samples (5-15μl) were transferred to microplate wells preloaded with ice cold PBS (200μl per well). Cell fluorescence was measured in high-throughput using the Attune™ NxT Acoustic Focusing Flow Cytometer / Attune NxT Autosampler system. Instrument settings were fixed at 440 FSC voltage (threshold value 1000), 440 SSC voltage (threshold value 1000) and 350 BL1 green voltage. Data acquisition settings were set at 50μl acquisition volume, 25μl/min flow rate and targeted 12,000 events recording per sample.

The FlowJo™ v10 software was used to analyse raw data acquired from flow cytometry. Data of recorded events were gated (rectangular gate) on a FSC-A/SSC-A plot to include more than 80% of recorded events for selecting *E. coli* TOP10 cell populations. Then, the gated *E. coli* TOP10 populations were analysed for levels of GFP expression (BL1-H) and to calculate the mean cell fluorescence value for each sample. A control cell line (I13504/pSB1C3 *E. coli* TOP10) was used to determine *E. coli* TOP10 cell mean autofluorecence value.

The derived mean cell fluorescence values were analysed to determine GFP output and response function for each AHL-receiver device (see "Data analysis methodology" in the Methods Section).

Supplementary Note 1 - Device metrics for use in genetic circuit designs

The metrics for genetic devices derived from characterisation experiments such as the activation fold change and EC50-value (Supplementary Figure 1) are important in assessing the device suitability for use in genetic circuit designs. The fold activation metric can be of significance when using these devices with downstream systems that require overcoming specific thresholds for activation/inhibition, e.g. in logic gate design. At the same time, the EC50 value can be of importance for engineering bacterial populations to respond to cell population densities.

Supplementary Note 2 - Characterisation of members of the Anderson promoter library

The Anderson collection is a library of constitutive *E. coli* promoters. The members of this collection show a wide range of transcriptional activities and have been very popular within the synthetic biology community as they enable straightforward fine tuning of gene expression levels. The J23101 promoter, a member of this library, frequently serves as the reference standard against which the transcription output (strength) of other promoters is benchmarked[1,2]. Calibration against a reference standard allows the straight-forward re-use of a promoter (or another device that makes use of a calibrated promoter) in other genetic designs as this method has been demonstrated to minimise variance in measured activity that results from the use of various cell hosts or experimental conditions[1]. To enable cross-correlating their activity to various other developed technologies[3-5] and their easier integration into other designs, we characterised the activity of the AHL-receiver devices relative to that of the J23101 promoter (Figure 3 in Main Text & Supplementary Table 1). Also, we characterised the relative strength of members of the Anderson collection that show a large variation in promoter strength as determined by flow cytometry (Supplementary Figure 2), since, to the best of our knowledge, this has not been documented elsewhere in the literature. These promoters have been characterised using a GFP expression reporter module (Bba_I13504 – Registry of Standard Biological Parts) on a pSB1C3 vector backbone using the characterisation protocol described in the "Materials and Methods" section in the main text.

Supplementary Note 3 – Software tool

Problem definition:

We obtained from experimental characterisation a data file of measurements from quorum sensing components. The data contain measurements from *n = 6* AHL-receiver devices and *m = 6* AHL inducer molecules. For each pair of device/AHL-inducer, the response of the device to the concentration of AHL is measured using a fluorescent reporter. Using these experimentally measured dose-responses, a fit to a four-parameter logistical dose-response curve was obtained.

Our goal is to process this dataset of fitted dose-response curves with an algorithm that finds orthogonal communication channels (pairs of device-AHL-inducers that will not react to each other according to the user-specified constraints of minimal activation threshold for specific gene expression, maximal activation threshold for non-specific gene expression, and number of chemical communication channels required).

Mathematical representation:

The aforementioned problem can be viewed as an instance of the combinatorial Rook Problem. In the Rook problem, we consider a chessboard with *n* rows, *m* columns and *k* rooks, where $2 \leq k \leq min(m,n)$. The task is to place all *k* rooks on the board in non-attacking positions. This is equivalent to distributing the rooks in such a way that in any given row and column there is only one rook placed. Supplementary Figure 3 shows two possible solutions for *n = 8*, *m = 8* and *k = 8*. The number of possible solutions, $R_{n,m,k}$, for a board with *n* rows and *m* columns on which *k* rooks need to be placed in non-attacking positions is given by:

$$R_{n,m,k} = \binom{n}{k}\binom{m}{k}k!$$

(Supplementary Equation 1)

If we assume that we have *n = 6* rows and *m = 6* columns (the number AHL-receiver devices and AHL-inducers described by the dataset in this study), then Supplementary Figure 4 shows the number of possible solutions calculated from Supplementary Equation 1.

Implementation of the algorithm:

The algorithm, implemented in Matlab® and summarised in Supplementary Figure 5, has two main parts. In the first part, which is executed only once per run, the data file is read along with the user-defined specifications for practical orthogonal communication (i.e. minimum fold change for specific gene expression and maximum fold change for non-specific gene expression). Then, the algorithm generates a data matrix (of dimension $n \times m$) where each coordinate stores the information about the given device-AHL pair. In the next step, the algorithm generates all possible channel combinations (possible placements of the rooks on the board), which are then evaluated in the second part.

In the second part, the algorithm reads one channel combination (e.g. [1-A, 3-C] for two communication channels) and evaluates it in terms of the given user-defined orthogonal

communication specifications. If the channel combination passes the evaluation process, then the algorithm finds a solution and stops. Otherwise, the algorithm reads the next combination and runs the evaluation process again. Finally, if all possible combinations have been evaluated but no solution that meets the specifications was found, then the algorithm exits with no solution. If this is the case, the user is advised to "relax" the constraining specifications. The accompanying Matlab® implementation of the algorithm in its default setting computes a single solution to the problem. Additionally, a provided switch functionality to a second setting allows the computation of all possible solutions for a given specification and data file.

Computational complexity analysis:

The computational complexity of the algorithm in the first part is dominated by the generation of possible combinations for the channels. Fortunately, a solution of the Rook Problem can be found in polynomial time using bipartite graph matching. The other steps involve reading a data file and calculating dose-response curves, which, in comparison, can be neglected in terms of their contribution to the computational complexity of the algorithm.

If we have $R_{n,m,k}$ possible solutions and consider a linear search to find a solution that satisfies user-defined specification, then, in the worst case scenario, the second part of the algorithm will have to run $R_{n,m,k}$ times. During each run, channel combinations are evaluated, which is a binary AND operation between signal and crosstalk data. Therefore, the second part can be assumed to have linear time complexity.

Runtime tests:

The performance of the algorithm on our dataset was tested 100 times. In each run, we generated a list of possible channel combinations for a given channel number, then randomised the order of the list. Runtimes for these trial runs are summarised in Supplementary Table 2.

Supplementary Figures

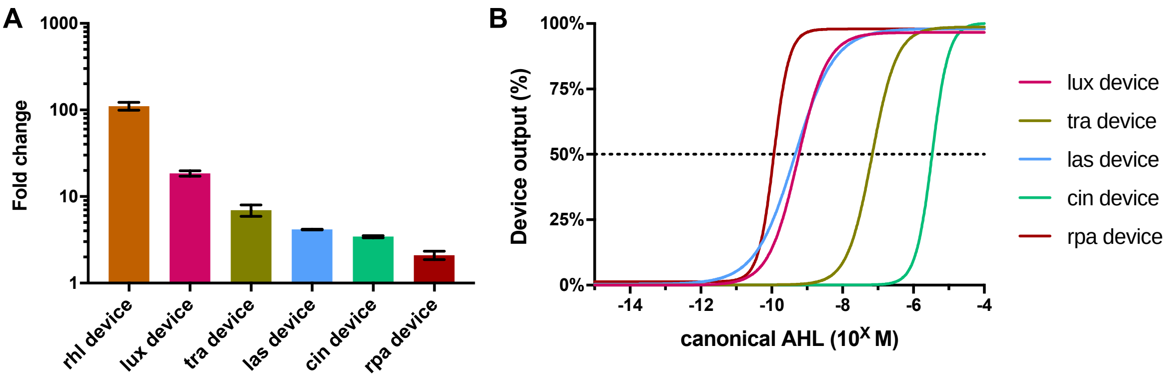

Supplementary Figure 1: AHL-receiver devices fold change and EC50. [A] Activation fold change [maximum expression/basal expression]. Error bars represent the standard error of the mean (SEM) for three biological replicates. **[B]** EC50 value (right) for AHL-receiver devices induced with cognate AHL. Input/output curves were derived from model fitting to the biological data for each AHL-responder device induced with its cognate AHL molecule.

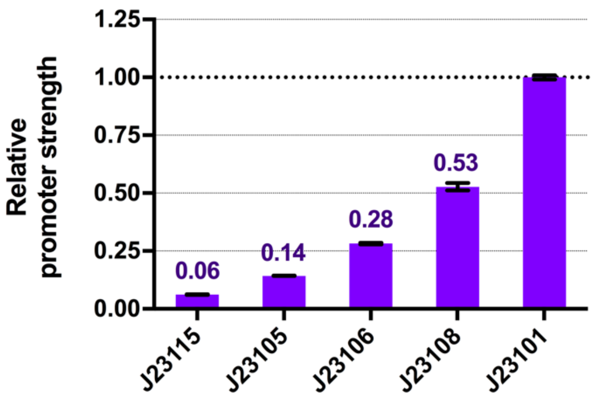

Supplementary Figure 2: Relative promoter strength of Anderson promoters. Relative promoter strength of constitutive promoters from the Anderson collection. Their relative strength has been determined against the output of the reference standard promoter (Bba_J23101) using the Bba_I13504 reporter module (rbs-GFP-double terminator) on a pSB1C3 vector backbone and by the use of flow cytometry (**Methods**). The dotted line indicates output of the reference standard promoter Bba_J23101. Error bars indicate the standard error of the mean for three biological replicates.

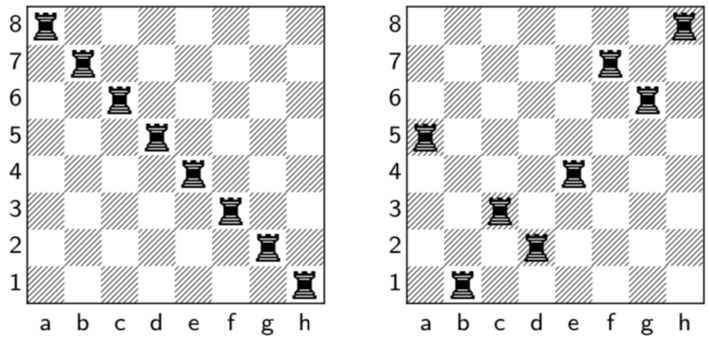

Supplementary Figure 3: The combinatorial Rook problem. In the Rook problem, we consider a chessboard with *n* rows and *m* columns. The task is to place all *k* rooks on the board in non-attacking positions. This amounts to be able to distribute the rooks in such a way that in any given row and column there is only one rook placed. Above, two possible solutions for *n = 8*, *m = 8* and *k = 8*.

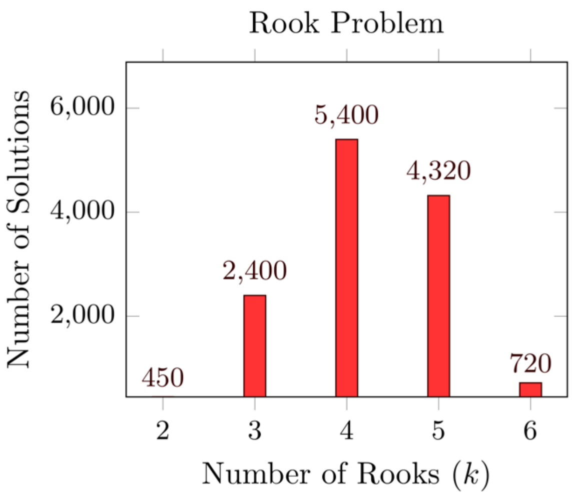

Supplementary Figure 4: Number of possible solutions for a 6x6 board. Number of possible solutions to the problem of how many combinations (pairs) of AHL-receiver device/AHL-inducer are possible given six devices and six inducers for 2, 3, 4, 5 or 6 chemical communication channels.

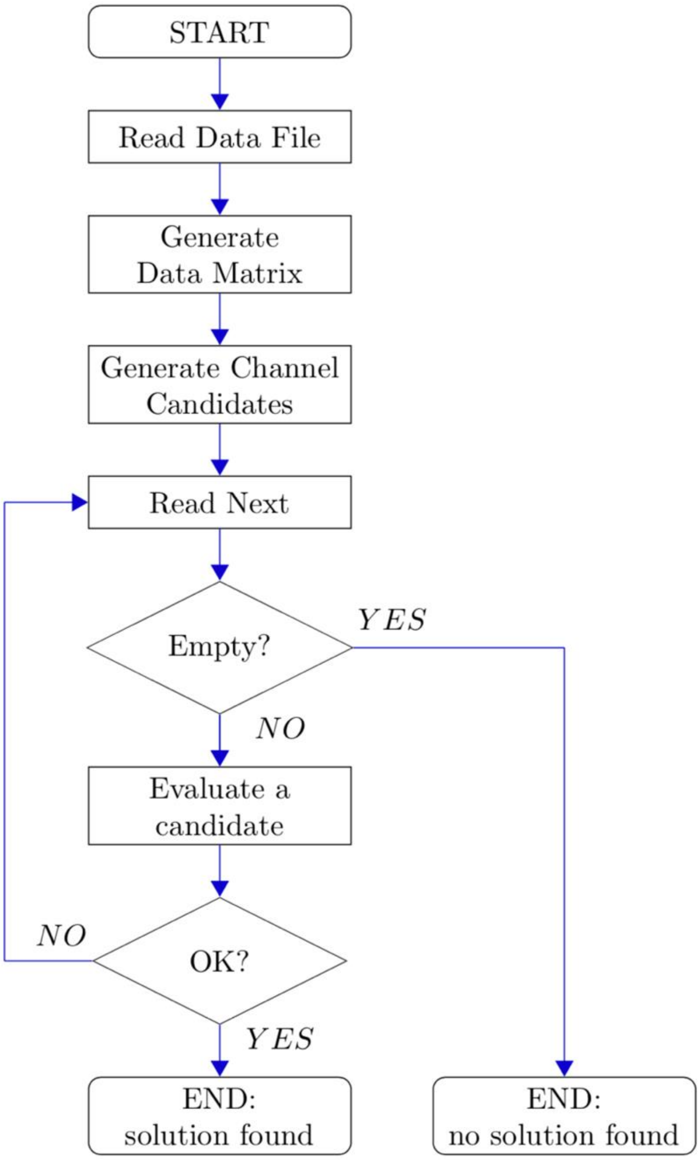

Supplementary Figure 5: Flowchart of the algorithm.

Supplementary Tables

Supplementary Table 1: Relative promoter strength of AHL-receiver devices. Relative promoter strength was determined against the output of the reference standard promoter (Bba_J23101) and by use of the Bba_I13504 reporter module (rbs-GFP-double terminator) on a pSB1C3 vector backbone. These promoter strengths were determined by flow cytometry using 1x10^{-15} M (low-state, red) and 1x10^{-4} M (high state, green) AHL concentrations (see the "Materials and Methods" section in the main text for methodology and data analysis).

Relative promoter strength

AHL-receiver device	C4 HSL		3O-C6 HSL		3O-C8 HSL	
	1x10^{-15} M	1x10^{-4} M	1x10^{-15} M	1x10^{-4} M	1x10^{-15} M	1x10^{-4} M
rhl device	0.00	0.12	0.00	0.09	0.00	0.05
lux device	0.09	0.36	0.09	1.53	0.09	1.65
tra device	0.00	0.00	0.00	0.03	0.00	0.03
las device	0.02	0.03	0.02	0.05	0.02	0.07
cin device	0.12	0.12	0.12	0.12	0.12	0.12
rpa device	0.87	0.97	0.87	1.60	0.87	1.47

AHL-receiver device	3O-C12 HSL		3OH-C14 HSL		pC HSL	
	1x10^{-15} M	1x10^{-4} M	1x10^{-15} M	1x10^{-4} M	1x10^{-15} M	1x10^{-4} M
rhl device	0.00	0.02	0.00	0.00	0.00	0.00
lux device	0.09	1.56	0.09	0.52	0.09	0.68
tra device	0.00	0.03	0.00	0.01	0.00	0.00
las device	0.02	0.07	0.02	0.07	0.02	0.04
cin device	0.12	0.20	0.12	0.41	0.12	0.11
rpa device	0.87	1.07	0.87	1.36	0.87	1.84

Supplementary Table 2: Runtime tests for orthogonal communication channels algorithm. The table shows the statistics of the runtime of the software, which was calculated from 100 runs. The table is divided into three sections. In the top section of the table, runtimes for the generation of possible combinations are shown. In the second section of the table, runtimes to find a solution, satisfying all design requirements of activation and crosstalk thresholds of 2-fold change are shown. The third section shows the runtime for finding all possible solutions for the same specification (which is the worst-case scenario of the algorithm, as explained in the complexity analysis section). Hardware specifications: MacBook Pro 2017 system, 3.1 GHz Intel Core i5 processor, 8 GB 2133 MHz LPDDR3 RAM.

**Number of channels**	2	3	4	5	6
**Possible Combinations**	450	2400	5400	4320	720
**List Generation (sec)**	0.0097	0.0333	0.0810	0.1184	0.1253
**First solution**					
avg. (sec)	0.0012	0.0054	0.0294		
min (sec)	0.0004	0.0034	0.0256		
max (sec)	0.0102	0.0188	0.0391		
**All solutions**					
**valid solutions**	48	28	3	0	0
avg. (sec)	0.1030	0.8582	2.6778	2.7470	0.5975
min (sec)	0.0870	0.7895	2.4653	2.5321	0.5260
max (sec)	0.2350	1.0508	3.5720	3.4553	0.8740

Supplementary Table 3: Plasmid constructs Plasmid constructs used in this study. Annotated DNA sequence files (.gb) can be found in the set of files accompanying this article

**Sequence name**	**Description**
cin device	Plasmid construct includes the cin system AHL-receiver device
cin device composite	Plasmid construct includes the cin system AHL-receiver device and a downstream GFP reporter module
las device	Plasmid construct includes the las system AHL-receiver device
las device composite	Plasmid construct includes the las system AHL-receiver device and a downstream GFP reporter module
lux device	Plasmid construct includes the lux system AHL-receiver device
lux device composite	Plasmid construct includes the lux system AHL-receiver device and a downstream GFP reporter module
rhl device	Plasmid construct includes the rhl system AHL-receiver device
rhl device composite	Plasmid construct includes the rhl system AHL-receiver device and a downstream GFP reporter module
rpa device	Plasmid construct includes the rpa system AHL-receiver device
rpa device composite	Plasmid construct includes the rpa system AHL-receiver device and a downstream GFP reporter module
tra device	Plasmid construct includes the tra system AHL-receiver device
tra device composite	Plasmid construct includes the tra system AHL-receiver device and a downstream GFP reporter module

Supplementary Table 4: Parameters from fitted logistical models for AHL-receiver devices/AHL-inducers library

Device	AHL	Basal expression	Maximum expression	Hill coefficient	EC50	Constraints and Notes	Fold Change
rhl device	C4 HSL	18.43	3278.22	0.64	4.68E-05	Bottom = 18.4	177.87
	3O C6 HSL	18.43	1881.91	0.78	1.62E-05	Bottom = 18.4	102.11
	30 C8 HSL	18.43	1108.84	0.89	2.25E-05	Bottom = 18.4	60.17
	3O C12 HSL	18.43	405.47	1.06	3.17E-05	Bottom = 18.4	22.00
	3OH C14	18.43	18.43	1.00	1.00E+00	Bottom = 18.4; Not converged	1.00
	pC HSL	18.43	75.83	1.82	1.15E-05	Bottom = 18.4	4.11
lux device	C4 HSL	1534.73	6321.03	4.59	2.17E-05	Bottom = 1535; Ambiguous	4.12
	3O C6 HSL	1534.73	28437.52	1.05	5.49E-10	Bottom = 1535	18.53
	30 C8 HSL	1534.73	29118.91	0.85	2.99E-09	Bottom = 1535	18.97
	3O C12 HSL	1534.73	26330.85	0.72	3.07E-08	Bottom = 1535	17.16
	3OH C14	1534.73	10139.72	1.28	2.18E-05	Bottom = 1535	6.61
	pC HSL	1534.73	13197.68	1.03	2.27E-07	Bottom = 1535	8.60

tra device	C4 HSL	79.73	79.73	1.00	1.00E+00	Bottom = 79.7; Top < 632; 0 < HillSlope < 2; Not converged	1.00
	3O C6 HSL	79.73	572.65	1.55	6.31E-08	Bottom = 79.7; Top < 632; 0 < HillSlope < 2	7.18
	30 C8 HSL	79.73	555.39	1.23	6.75E-08	Bottom = 79.7; Top < 632; 0 < HillSlope < 2	6.97
	3O C12 HSL	79.73	600.68	0.87	1.39E-05	Bottom = 79.7; Top < 632; 0 < HillSlope < 2	7.53
	3OH C14	79.73	154.32	2.00	4.22E-05	Bottom = 79.7; Top < 632; 0 < HillSlope < 2; Ambiguous	1.94
	pC HSL	79.73	79.73	1.00	1.00E+00	Bottom = 79.7; Top < 632; 0 < HillSlope < 2; Not converged	1.00
las device	C4 HSL	302.4	460.82	0.40	1.32E-06	Bottom = 302; Top < 1255	1.52
	3O C6 HSL	302.4	1255.00	0.78	4.93E-05	Bottom = 302; Top < 1255; Hit constrain	4.15
	30 C8 HSL	302.4	1205.84	0.61	2.46E-07	Bottom = 302; Top < 1255	3.99
	3O C12 HSL	302.4	1254.03	0.76	4.38E-10	Bottom = 302; Top < 1255	4.15
	3OH C14	302.4	1255.00	0.56	1.46E-07	Bottom = 302; Top < 1255; Hit constrain	4.15
	pC HSL	302.4	1255.00	0.57	1.73E-04	Bottom = 302; Top < 1255; Hit constrain	4.15
cin device	C4 HSL	2081.73	2116.96	-1.15	2.64E-06	Bottom = 2082; Top < 7128; LogEC50 > -5.58; HillSlope < 2; Hit constraint	1.02

	3O C6 HSL	2081.73	7128.00	2.00	9.18E-04	Bottom = 2082; Top < 7128; LogEC50 > -5.58; HillSlope < 2; Ambiguous	3.42
	30 C8 HSL	2081.73	2176.68	2.00	7.69E-06	Bottom = 2082; Top < 7128; LogEC50 > -5.58; HillSlope < 2; Hit constraint	1.05
	3O C12 HSL	2081.73	3543.80	1.63	2.65E-06	Bottom = 2082; Top < 7128; LogEC50 > -5.58; HillSlope < 2	1.70
	3OH C14	2081.73	7126.34	1.87	3.41E-06	Bottom = 2082; Top < 7128; LogEC50 > -5.58; HillSlope < 2	3.42
	pC HSL	2081.73	2081.73	1.00	1.00E+00	Bottom = 2082; Top < 7128; LogEC50 > -5.58; HillSlope < 2; Not converged	1.00
rpa device	C4 HSL	15208.06	15208.06	1.00	1.00E+00	Bottom = 15208; Top < 32016; HillSlope < 2; Not converged	1.00
	3O C6 HSL	15208.06	27992.27	1.86	8.11E-06	Bottom = 15208; Top < 32016; HillSlope < 2	1.84
	30 C8 HSL	15208.06	32016.00	1.15	6.43E-05	Bottom = 15208; Top < 32016; HillSlope < 2; Hit constrain	2.11
	3O C12 HSL	15208.06	18938.06	2.00	2.74E-05	Bottom = 15208; Top < 32016; HillSlope < 2	1.25
	3OH C14	15208.06	32012.15	2.00	9.80E-05	Bottom = 15208; Top < 32016; HillSlope < 2; Ambiguous	2.10
	pC HSL	15208.06	32016.00	1.76	1.14E-10	Bottom = 15208; Top < 32016; HillSlope < 2; Hit constrain	2.11

Supplementary References

1 Kelly, J. R. *et al.* Measuring the activity of BioBrick promoters using an in vivo reference standard. *J Biol Eng* **3**, 4, (2009).

2 Rudge, T. J. *et al.* Characterization of Intrinsic Properties of Promoters. *ACS Synth Biol* **5**, 89-98, (2016).

3 Fernandez-Rodriguez, J., Moser, F., Song, M. & Voigt, C. A. Engineering RGB color vision into Escherichia coli. *Nat Chem Biol* **13**, 706-708, (2017).

4 Schaerli, Y., Gili, M. & Isalan, M. A split intein T7 RNA polymerase for transcriptional AND-logic. *Nucleic Acids Res* **42**, 12322-12328, (2014).

5 Pasini, M. *et al.* Using promoter libraries to reduce metabolic burden due to plasmid-encoded proteins in recombinant Escherichia coli. *N Biotechnol* **33**, 78-90, (2016).
