# Supplementary material for: Tools for engineering coordinated system behaviour in synthetic microbial consortia
Source: Nat Commun. 2018 Jul 11;9:2677. doi: 10.1038/s41467-018-05046-2 (PMC6041260; doi:10.1038/s41467-018-05046-2)
Supplement: Supplementary file 3 — Description of Additional Supplementary Files [file 41467_2018_5046_MOESM3_ESM.pdf]

Description of Additional Supplementary Files

File Name: Supplementary Software
Description: A CAD tool for the automatic identification of non-interfering (orthogonal) chemical channels for the design of cell-to-cell communication systems.
